# Effects of low-calorie and different weight-maintenance diets on IgG glycome composition

**DOI:** 10.3389/fimmu.2022.995186

**Published:** 2022-09-21

**Authors:** Helena Deriš, Petra Tominac, Frano Vučković, Nina Briški, Arne Astrup, Ellen E. Blaak, Gordan Lauc, Ivan Gudelj

**Affiliations:** ^1^Genos Glycoscience Research Laboratory, Zagreb, Croatia; ^2^Centre for Healthy Weigh, The Novo Nordisk Foundation, Hellerup, Denmark; ^3^Department of Human Biology, NUTRIM, School for Nutrition and Translational Research in Metabolism, Maastricht University, Maastricht, Netherlands; ^4^Faculty of Pharmacy and Biochemistry, University of Zagreb, Zagreb, Croatia; ^5^Department of Biotechnology, University of Rijeka, Rijeka, Croatia

**Keywords:** IgG, glycosylation, calorie reduction, weight maintenance, healthy diet

## Abstract

Obesity-induced inflammation activates the adaptive immune system by altering immunoglobulin G (IgG) glycosylation in a way to produce more proinflammatory antibodies. The IgG glycome has already been well studied, and its alterations are correlated with a high body mass index (BMI) and central adiposity. Still, the IgG N-glycome susceptibility to different dietary regimes for weight control after the initial weight loss has not been studied. To explore changes in IgG glycosylation induced by weight loss and subsequent weight-maintenance diets, we analyzed 1,850 IgG glycomes from subjects in a dietary intervention Diogenes study. In this study, participants followed a low-calorie diet (LCD) providing 800 kcal/d for 8 weeks, followed by one of five weight-maintenance diets over a 6-month period. The most significant alteration of the IgG N-glycome was present 8 weeks after the subjects underwent an LCD, a statistically significant decrease of agalactosylated and the increase of sialylated N glycans. In the follow-up period, the increase in glycans with bisecting GlcNAc and the decrease in sialylated glycans were observed. Those changes were present regardless of the diet type, and we did not observe significant changes between different diets. However, it should be noted that in all five diet groups, there were individuals who prominently altered their IgG glycome composition in either proinflammatory or anti-inflammatory directions.

## Introduction

Being overweight or obese is associated with numerous non-communicable and infectious (including Coronavirus disease 2019 (COVID-19) (1)) diseases, yet overweight/obesity is on its, seemingly, unstoppable global rise; over 2 billion people have excess body weight, which is over a quarter of the world population (2). Moreover, the global study on over 10.6 million participants showed that both overweight [body mass index (BMI) ≥ 25 kg/m^2^] and obesity (BMI ≥ 30 kg/m^2^) are associated with increased all-cause mortality (3). Given these overweight/obesity-related associations, weight loss is often a target that attenuates inflammation and reduces the risk of developing further health complications. Yet, losing weight is a more achievable goal than the maintenance of the reduced weight, which is crucial to achieving long-term health improvements (4).

Obesity-induced inflammation is often the cause of these comorbidities and eventually mortality; the inflammation usually starts with the activation of the innate immune system and consequently activates the adaptive immune system affecting multiple organs, from adipose tissue, pancreas, liver, and skeletal muscle to heart and brain (5). During this adaptive immune system activation, there is an accumulation of B cells in the adipose tissue, the production of a more inflammatory repertoire of cytokines and pathogenic immunoglobulin G (IgG) antibodies (6). Each IgG molecule has an N-glycosylation site on the Asn–297 of the constant heavy 2 (CH2) domain on each of its heavy chains, which influences its structural stability, conformation, and half-life, as well as effector functions (7). As with most cell surface and secreted proteins, IgG glycosylation varies widely depending on the inflammatory state. Unlike other proteins, these changes directly contribute to altered effector function and add an extra dimension to the functional diversity of antibodies (8). Under homeostatic conditions, the IgG glycome composition within an individual is stable over time. However, it changes gradually with age and can change rapidly in many homeostatic disorders. Significant changes in the IgG glycome have also been reported in several diseases such as cancer, autoimmune diseases, infectious diseases, and inflammatory diseases and conditions (7). By modulating its affinity for different FcγR receptors, the different terminal monosaccharides of N-glycans direct IgG binding to a preferential receptor and thereby effect its function. For example, a higher proportion of sialic acid and galactose residues is associated with anti-inflammatory activity, while the lack of sialic acid and galactose and presence of bisecting N-acetylglucosamine (GlcNAc) residues are linked with proinflammatory activity (7). Indeed, these proinflammatory IgG glycome traits have already been associated with a higher BMI and the measures of central adiposity (9, 10). It is also important to emphasize that not only IgG glycosylation is associated with chronic inflammation and associated diseases but also the glycosylation of other proteins (e.g., other plasma proteins), which indicates that glycan processing may be, *per se*, altered under these conditions (11).

A wide range of high-throughput analytical platforms can be used for the profiling, characterization, and analysis of IgG N-glycans. Liquid chromatography coupled with fluorescence detection (LC-FLR) is the most widespread (12). High-throughput sample preparation for the separation and detection of LC-FLR glycans usually begins with the enzymatic release of glycan moieties from the protein backbone, followed by the labeling of free glycans with tags containing fluorophores (13). The most used tags are 2-aminobenzamide and procainamide. Both labels use the same reductive amination mechanism to bind stoichiometrically to a glycan molecule, allowing for relative quantification based on Fluorescence (FLR) intensity. Recently, a new labeling compound was introduced, RapiFluor MS (RFMS). RFMS is an instant labeling agent containing a quinoline fluorophore for FLR detection and an n-hydroxysuccinimide group that binds to N-glycan-glycosylamine creating a stable urea linkage. Compared to traditional tags, RFMS enables fast N-glycan labeling and allows for better throughput, which comes in handy when larger numbers of samples are analyzed (12, 13).

To explore IgG glycosylation alterations due to weight loss and subsequent weight-maintenance diets, we analyzed 1,850 IgG glycomes in plasma from subjects of the Diogenes study (14, 15), one of the largest dietary intervention studies in which subjects underwent a low-calorie diet (LCD) followed by one of five weight-maintenance diets [low protein (LP)/low glycemic index (LGI), LP/high glycemic index (HGI), high protein (HP)/LGI, HP/HGI, and control] in a period of 6 months when subjects are vulnerable to regain the weight.

## Material methods

### Subjects

The glycome composition of IgG isolated from 1,850 plasma samples was analyzed from eight different centers of the Diogenes study, described elsewhere in detail (14, 15), at three different time points: at the beginning of the diet intervention (time point 1), after 8 weeks on an LCD diet providing 800 kcal/d (time point 2), and after 6 months on a weight-control diet (time point 3). Subjects on a diet according to current national dietary guidelines (“healthy diet”) in each of the countries served as the control group. After the initial screening, 938 subjects, 620 women and 312 men, with a mean age of 40 years for women and 42 years for men and a mean BMI of 33.8 kg m^-2^ for both sexes entered the LCD phase of the study. The mean waist circumference of the women who participated in the LCD intervention was 103.0 cm and that of men was 112.4 cm (14). A total of 773 participants who completed this phase with an achieved target weight loss of 8% of baseline body weight were randomly assigned to one of the five maintenance diets, of which only 548 participants completed the intervention (71%). Fewer participants in the HP and LGI groups than in the LP/HGI group discontinued from the study, 26.4% and 25.6% *vs*. 37.4%, respectively (16). In-depth information on the number of subjects and types of diets per each center is presented in **Supplementary Table 1**.

### Sample preparation, rapid rapifluor-MS N-glycan labeling and hydrophilic interaction chromatography–solid-phase extraction clean-up

Samples were randomly positioned into 26 96-well plates. Each 96-well plate contained approximately 70 samples, as well as five randomly chosen sample replicates from the same plate and five from other plates. IgG was isolated using 96-well protein G monolithic plates (BIA Separations, Ajdovscina, Slovenia) following a protocol first described by Pučić et al. (17) and Trbojević-Akmačić et al. (18). Briefly, plasma samples were first diluted and filtered through a 0.45 μm GHP filter plate (Pall Corporation, Port Washington, New York ,USA) and transferred to a protein G monolithic plate. Samples were then repeatedly washed with 1× phosphate-buffered saline (1× PBS) and eluted with 0.1 mol/L formic acid (Merck, Darmstadt, Germany) followed by an immediate neutralization with ammonium bicarbonate (Acros Organics, Waltham, Massachusetts, USA). An appropriate volume of isolated IgG (average mass of 15 µg) was dried in a vacuum centrifuge.

Further sample analysis (deglycosylation, released N-glycan labeling, and clean-up) was performed using the Waters GlycoWorks RapiFluor-MS N-Glycan Kit (USA) according to the Producer’s instructions (Waters Corporation, 2017), with some adaptations of the protocol to suit high-throughput analysis in the 96-well PCR plate format (19). In short, isolated and dried IgG was first resuspended in ultrapure water, swiftly denatured with a RapiGest SF surfactant solution, and enzymatically deglycosylated with GlycoWorks Rapid PNGase F. Released N-glycans were then labeled with the RapiFluor-MS fluorescent dye. Labeled IgG N-glycans were purified by hydrophilic interaction chromatography–solid-phase extraction (HILIC-SPE) cleanup by repeated washing steps with formic acid/ultrapure water/acetonitrile (ACN) (1:9:90, v/v/v) and eluted in three steps with an SPE elution buffer, 200 mmol/L ammonium acetate/ACN (95:5, v/v), pH 7. To dilute the samples, a sample diluent, dimethylformamide/ACN (32:68, v/v) was added to each sample and mixed. Diluted samples were either immediately used in further chromatographic analysis or were stored at -20°C until further use.

### Hydrophilic interaction chromatography–ultra-high-performance liquid chromatography with fluorescence detection N-glycan analysis

The analysis of RapiFluor-MS-labeled IgG N-glycans was performed on Waters Acquity UPLC H-class instruments monitored by Waters Empower 3 software and using Waters UPLC Glycan bridged ethylene hybrid (BEH) amide chromatographic columns (130 Å, 1.7 µm BEH particles, 2.1 × 100 mm) with 50 mmol/L ammonium formate, pH 4.4 as solvent A, and 100% LC-MS grade ACN as solvent B, as previously described by Keser et al. (20). The adjustments of the separation method made by Deriš et al. (19) included a linear gradient of 75%–61.5% acetonitrile (v/v) at a flow rate of 0.4 ml/min over 30 min in a 42-min analytical run and an injection volume of 30 µl. Chromatograms acquired in the analysis were automatically integrated, separated into 22 glycan peaks (**Supplementary Figure 1**), and total area-normalized (%Area) values were obtained for each peak to enable a relative quantification of IgG N-glycans.

### Data analysis

Normalization and batch correction were performed on UHPLC glycan data to remove the experimental variation of measurements. To make the measurements across samples comparable, normalization by the total area was performed where the peak area of each of 22 glycan structures was divided by the total area of the corresponding chromatogram. Prior to batch correction, normalized glycan measurements were log-transformed due to the right-skewness of their distributions and the multiplicative nature of batch effects. The batch correction was performed on log-transformed measurements using the “ComBat” method (R package sva) where the technical source of variation was modeled as a batch covariate. Estimated batch effects were subtracted from log-transformed measurements to obtain measurement correction for experimental noise.

Six derived traits were calculated from 22 glycan structures directly obtained by UHPLC analysis. Derived glycan traits represent a portion of structurally similar glycan species with common biosynthetic pathways. The total IgG-derived glycan traits were calculated as the ratios of glycan peaks (GP1–GP22) with the same structural characteristics in a total IgG glycome: total agalactosylated glycans, G0 = (GP1 + GP2 + GP3 + GP4)/SUM (GP1-GP22)*100; total monogalactosylated glycans, G1 = (GP5 + GP6 + GP7 + GP8 + GP9 + GP10)/SUM (GP1-GP22)*100; total digalactosylated glycans G2 = (GP11 + GP12 + GP13)/SUM (GP1-GP22)*100; total sialylated glycans, S = (GP14 + GP15 + GP16 + GP17 + GP18 + GP19 + GP20 + GP21 + GP22)/SUM (GP1-GP22)*100; total fucosylated glycans F = (GP1 + GP3 + GP4 + GP7 + GP8 + GP9 + GP10 + GP12 + GP13 + GP14 + GP16 + GP17 + GP21 + GP22)/SUM (GP1-GP22)*100; total glycans with bisecting GlcNAc, B = GP4 + GP9 + GP10 + GP13 + GP17 + GP20 + GP22)/SUM (GP1-GP22)*100.

The longitudinal analysis of patient samples through their observation period was performed by implementing a linear mixed-effects model where glycan measurement was the dependent variable and time was modeled as a fixed effect, while individual ID was included in the model as a random intercept, with age, gender, and BMI included as additional covariates. The analyses were firstly performed for each center separately and then combined using a random effects meta-analysis approach (R package meta, metagen (method = “ML”)). Prior to the analyses, glycan variables were all transformed to standard normal distribution (mean=0, sd=1) by the inverse transformation of ranks to Normality (R package “GenABEL”, function rntransform). The usage of rank-transformed variables in analyses makes the estimated effects of different glycans in different centers comparable as transformed glycan variables have the same standardized variance. The false discovery rate was controlled using the Benjamini–Hochberg procedure (function p.adjust(method = “BH”)). Data were analyzed and visualized using R programming language (version 3.0.1).

## Results

The N-glycome composition of IgG isolated from subjects’ plasma samples was determined by the UHPLC analysis of glycans labeled with RapiFluor-MS as described in the Materials and Methods section. Statistical analysis was performed on six derived traits calculated from 22 directly measured glycan structures, corresponding to 22 glycan peaks obtained by UHPLC analysis.

**Figure 1:**
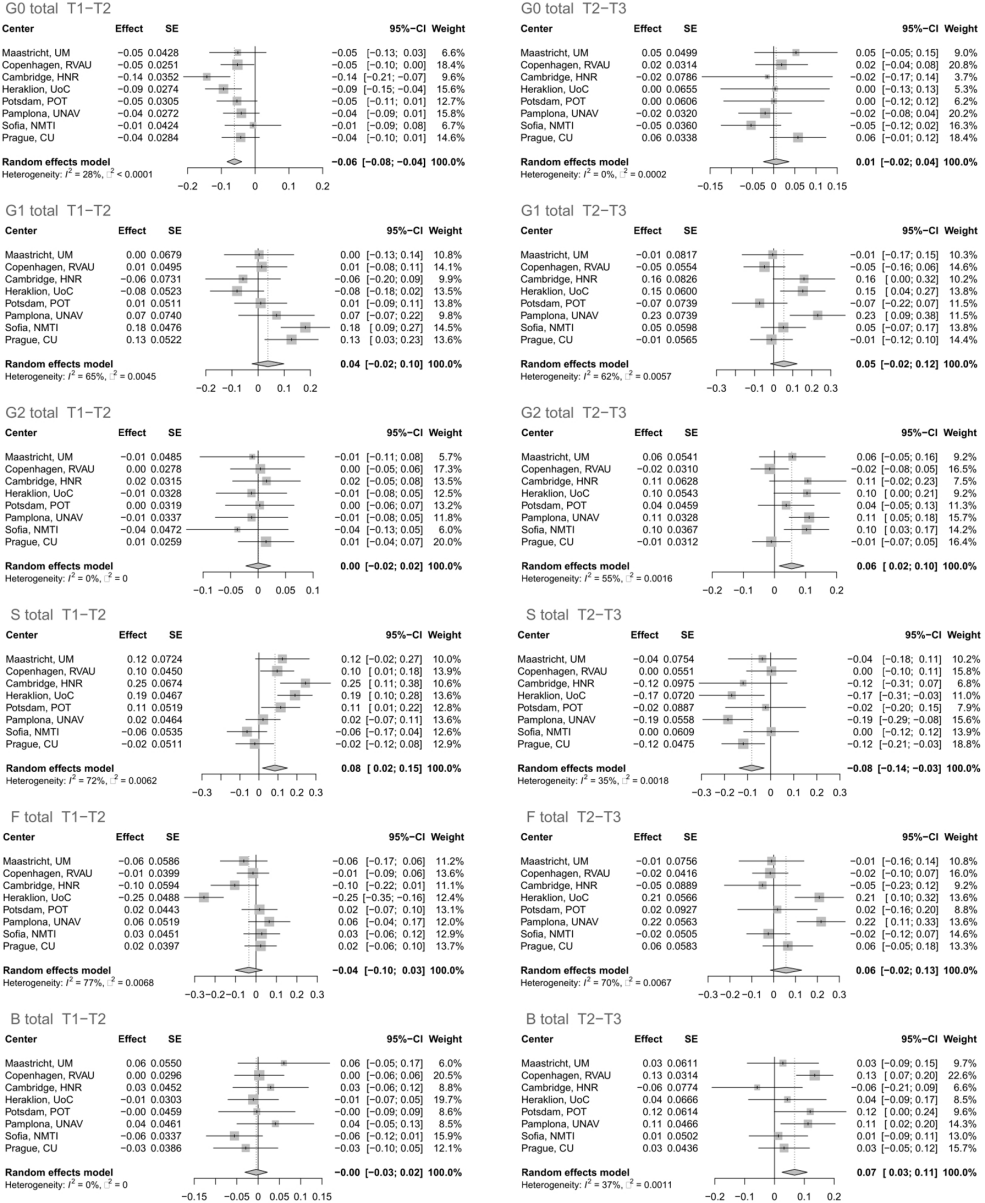
Effect of calorie loss on the immunoglobulin G (IgG) glycome in the first time period (T1–T2) and effect of different weight maintenance diets on the IgG glycome in the second time period (T2–T3). Changes in IgG glycome composition after performing meta-analysis for all the centers are shown. SE, standard error; 95% CI, 95% confidence interval; T1, time point 1; T2, time point 2; T3, time point 3; G0, agalactosylated glycans; G1, monogalactosylated glycans; G2, digalactosylated glycans; S, glycans containing sialic acid; F, glycans with core fucose; B, glycans with bisecting GlcNAc.

**Figure 2:**
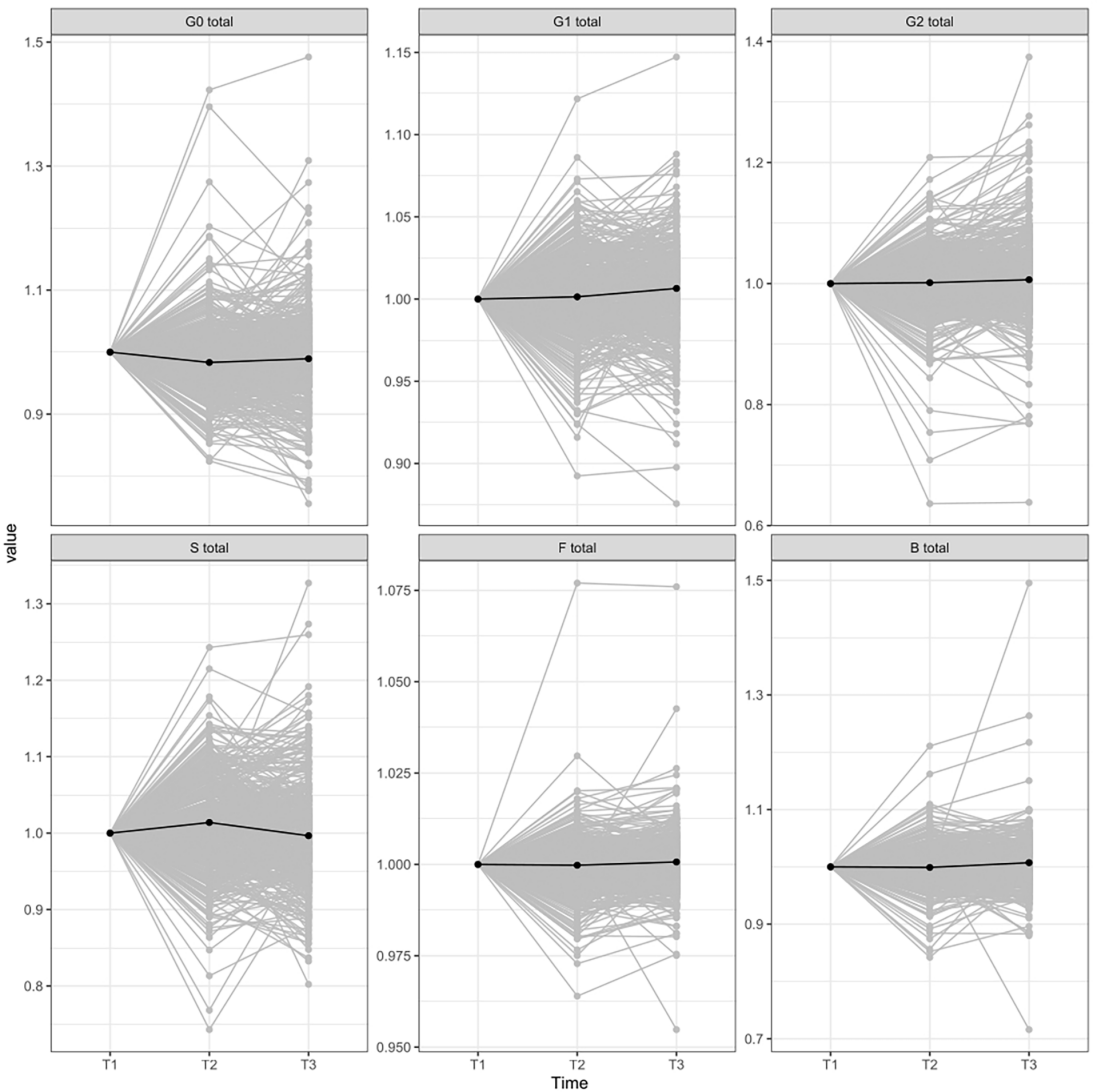
IgG glycome composition changes between different time-periods normalized to the first point. T1, time point 1; T2, time point 2; T3, time point 3; G0, agalactosylated glycans; G1, monogalactosylated glycan structures; G2, glycans with two galactoses; S, glycans containing sialic acid; F, glycans with core fucose; B, glycans with bisecting GlcNAc.

The glycan analysis was firstly performed for each center of the Diogenes study separately on rank-transformed glycan variables (**Supplementary Table 2**; **Supplementary Figure 2**). After performing meta-analysis for all the centers, four derived traits showed statistically significant alterations in their levels, either in the first (T1–T2) or second (T2–T3) time period (**Figure 1**). The most significant change after meta-analysis in the first time period, representing an 8-week long period on LCD with a weight loss of ~11 kg, is a decrease of agalactosylated structures (adjusted p-value < 0.001) accompanied by an increase of sialylated structures (adjusted p-value < 0.03) (**Supplementary Table 3**; **Figure 2**). On the other hand, in the second time period with weight maintenance, there was a statistically significant increase in the abundance of digalactosylated structures and structures with bisecting GlcNAc (adjusted p-value < 0.02), while sialylated glycans went to the opposite direction when compared to the first time period (adjusted p-value < 0.02) (**Supplementary Table 3**; **Figure 2**).

Additionally, we did not observe any significant changes in derived traits between different weight-control diets (**Supplementary Figure 3**), nor did we find any correlation between IgG N-glycan traits and age, sex, and BMI after the LCD period or after the second, the weight maintenance period (**Supplementary Table 4**).

## Discussion

The Diogenes dietary intervention study deepened our understanding of the impact of the weight-loss and weight-maintenance diets on the adipose tissue (AT) transcriptome (21), different clinical parameters [e.g., blood pressure (BP) (22) and fasting insulin and glucose (23)], blood proteome, and steroid hormones (4), and this is now further extended by IgG glycome analysis. The IgG glycome has already been well studied, and its alterations are associated with different diseases and conditions, including high BMI and central adiposity (7, 9, 10), yet this is the first study that shed light on its susceptibility to different dietary regimes for weight control after weight loss.

The most prominent alteration of the glycome was observed 8 weeks after the subjects underwent LCD with a weight loss of approximately 11 kg; a statistically significant decrease of agalactosylated N-glycans and an accompanying increase of sialylated N-glycans in the IgG glycome present shifting from pro- to the anti-inflammatory activity of IgG (7). These observations are expected knowing that adipose tissue, apart from being an energy storage depot, is also an endocrine organ that produces adipokines such as interleukin (IL-6) and tumor necrosis factor-α (TNF-α), which are cytokines already well linked with inflammation (24, 25). Since their adipose tissue production is affected by the degree of adiposity, the attenuation of systematic inflammation is an obvious outcome after a weight-reducing energy-restricted diet. Indeed, both of these cytokines and the attenuation of inflammation have been associated with the alteration of the IgG glycome (7). Thus, gene and protein association studies have found a correlation between IL-6 and proinflammatory IgG glycosylation patterns in humans (26, 27), while TNF-α serum levels are correlated with a proinflammatory IgG glycome pattern that is shown to be susceptible to anti-TNF-α therapy (28–30). Unfortunately, aside from these association studies, there is no study that illuminated the underlying mechanism in which inflammation modulates IgG glycosylation. However, our study is not the first one that observed an increase of anti-inflammatory IgG glycan repertoire after LCD (31). The IgG glycome changes toward the anti-inflammatory direction were reported just after a week of fasting, yet they did not correlate significantly with the clinical improvement after the vegetarian diet period (31).

The mean weight gain in 548 participants who completed the 6-month intervention was 0.565 ± 44 kg. Only the participants assigned to the LP/HGI diet had a significant weight gain of approximately 1.7 kg. Body weight changes differed between the diet groups, and each group did not differ significantly in terms of diet-related adverse events (16). Similarly, we did not observe significant changes between different diets during the weight-maintenance period, and an increased abundance of glycans with bisecting GlcNAc and a decrease of sialylated ones, which were present regardless of the diet type, suggest the proinflammatory direction of IgG glycome alteration in the period of weight maintaining or regaining.

Therefore, our study together with the study published by Kjeldsen-Kragh et al. suggests that calorie intake and weight loss rather than a diet type is the main driver of IgG glycome changes (31). Berry et al. also published a study where they challenged the logic of the standardized, universal diet and recommended personalized nutrition for disease prevention (32), and the IgG glycome alterations we observed seem to support this claim. Interestingly, this distinguishes IgG glycosylation and its susceptibility to change from clinical and biochemical parameters observed/measured after a vegetarian diet or Diogenes diets (31, 33). Specifically, in rheumatoid arthritis patients’ IgG glycome, significant changes were observed already after 7–10 days after fasting and there were no significant changes after following a vegetarian diet even though clinical improvement was reported also after the vegetarian diet period (31). Similarly, of the Diogenes study diets, the HP/LGI diet was associated with reduced body fatness and beneficial effects on blood pressure, blood lipids, and inflammation (33), yet this diet’s beneficial influence had no impact on the IgG glycome despite its association with all of these clinical/biochemical parameters (7).

In addition, a comprehensive evaluation of several antiaging diets, published by Lee et al. (34), revealed how calorie reduction and similar diets (including fasting, time-restricted feeding, and ketogenic diet), may not always be uniformly beneficial in extending a life span and their outcome is highly dependent on the genotype. Moreover, they concluded that a ubiquitous endorsement of a particular dietary intervention for healthy longevity would be impractical, which strongly coincides with our findings regarding the influence of dietary composition on IgG glycosylation.

In conclusion, this study is limited by the types of included diets and the number of measured biochemical parameters and consequently unable to illuminate the link between the IgG glycome and adipose tissue inflammation. However, by analyzing 1,850 IgG glycomes from subjects following one of five different diets and finding no association between glycome alterations and diet types, this study reinforces transition from a universal, standardized to a personalized nutrition approach for the achievement of overall health benefits and the need for further research of relations between obesity, inflammation, and glycosylation.

## Data availability statement

The original contributions presented in the study are included in the article/**Supplementary Material**. Further inquiries can be directed to the corresponding authors.

## Ethics statement

The studies involving human participants were reviewed and approved by the local ethics committees in the respective countries. The protocol was in accordance with the Declaration of Helsinki; all study participants signed an informed consent document after they received verbal and written instructions and according to local legislation. The ethics committee waived the requirement of written informed consent for participation.

## Author contributions

HD, NB and PT carried out the experiments. FV was involved in data analysis and interpretation, and visualization. AA, EB and GL were involved in funding acquisition, study design, and reviewing and editing the manuscript. HD and IG were involved in original draft preparation, reviewing, and editing, and IG supervised the project. All authors contributed to the article and approved the submitted version.

## Funding

This work was supported by the European Structural and Investment Funds grant for the Croatian National Centre of Competence in Molecular Diagnostics [grant number KK.01.2.2.03.0006]; IRI “CardioMetabolic” grant [grant number KK.01.2.1.02.0321]; and Croatian National Centre of Research Excellence in Personalized Healthcare grant [grant number KK.01.1.1.01.0010].

## Acknowledgments

This work was supported by the Human Glycome Project. Equipment and products from Waters and New England Biolabs^®^, Inc. were used for this research. The work of doctoral student Helena Deriš has been supported in part by the “Young researchers’ career development project – training of doctoral students” of the Croatian Science Foundation.

## Conflict of interest

GL is the founder and owner and HD, PT, FV and IG are employees of Genos Ltd, a company that specializes in high-throughput glycomics and has several patents in this field. Author AA was employed by The Novo Nordisk Foundation.

The remaining authors declare that the research was conducted in the absence of any commercial or financial relationships that could be construed as a potential conflict of interest.

## Publisher’s note

All claims expressed in this article are solely those of the authors and do not necessarily represent those of their affiliated organizations, or those of the publisher, the editors and the reviewers. Any product that may be evaluated in this article, or claim that may be made by its manufacturer, is not guaranteed or endorsed by the publisher.

## Abbreviations

LCD, low-calorie diet; LP, low protein; HP, high protein; LGI, low glycemic index; HGI, high glycemic index; U(H)PLC, ultra-(high-)performance liquid chromatography.
