# Supplementary material for: Effects of low-calorie and different weight-maintenance diets on IgG glycome composition
Source: Front Immunol. 2022 Sep 21;13:995186. doi: 10.3389/fimmu.2022.995186 (PMC9535357; doi:10.3389/fimmu.2022.995186)
Supplement: Supplementary file 1 [file DataSheet_1.docx]

Supplementary Information for

**Effects of low-calorie and different weight-maintenance diets on IgG glycome composition**

Helena Deriš^1^, Petra Tominac^1^, Frano Vučković^1^, Nina Briški^1^, Arne Astrup^2^, Ellen E. Blaak^3^, Gordan Lauc^1,4*^, Ivan Gudelj^1,5*^

^1^Genos Glycoscience Research Laboratory, Zagreb, Croatia

^2^Centre for Healthy Weigh, the Novo Nordisk Foundation, Hellerup, Denmark

^3^Dept of Human Biology, NUTRIM, School for Nutrition and Translational Research in Metabolism, Maastricht University, Maastricht, Netherlands

^4^University of Zagreb, Faculty of Pharmacy and Biochemistry, Zagreb, Croatia

^5^University of Rijeka, Department of Biotechnology, Rijeka, Croatia

*** Correspondence:**

Gordan Lauc

glauc@pharma.hr

Ivan Gudelj

ivan.gudelj@uniri.hr

Supplementary Table 1. Descriptive information on the number of subjects included in the study. T1 - time point 1, T2 - time point 2, T3 - time point 3, HP - high protein, LP - low protein, HGI - high glycaemic index, LGI - low glycaemic index.

| Center | Time  point | Samples (total = 1850) | | | | | |
| --- | --- | --- | --- | --- | --- | --- | --- |
|  |  | Healthy diet | HP / HGI | HP /LGI | LP /HGI | LP /LGI | total |
| Maastricht, UM | T1 | 22 | 22 | 20 | 30 | 19 | **113** |
|  | T2 | 23 | 21 | 19 | 30 | 20 | **113** |
|  | T3 | 17 | 14 | 19 | 17 | 17 | **84** |
|  | total | **62** | **57** | **58** | **77** | **56** |  |
| Copenhagen, RVAU | T1 | 23 | 26 | 23 | 22 | 27 | **121** |
|  | T2 | 23 | 27 | 23 | 22 | 27 | **122** |
|  | T3 | 17 | 20 | 19 | 15 | 20 | **91** |
|  | total | **63** | **73** | **65** | **59** | **74** |  |
| Cambridge, HNR | T1 | 14 | 13 | 13 | 15 | 12 | **67** |
|  | T2 | 15 | 13 | 13 | 15 | 12 | **68** |
|  | T3 | 8 | 7 | 10 | 3 | 6 | **34** |
|  | total | **37** | **33** | **36** | **33** | **30** |  |
| Heraklion, UoC | T1 | 13 | 12 | 16 | 14 | 12 | **67** |
|  | T2 | 13 | 11 | 15 | 14 | 12 | **65** |
|  | T3 | 6 | 8 | 9 | 3 | 5 | **31** |
|  | total | **32** | **31** | **40** | **31** | **29** |  |
| Potsdam, POT | T1 | 15 | 15 | 15 | 15 | 16 | **76** |
|  | T2 | 15 | 16 | 16 | 15 | 16 | **78** |
|  | T3 | 15 | 11 | 14 | 9 | 13 | **62** |
|  | total | **45** | **42** | **45** | **39** | **45** |  |
| Pamplona, UNAV | T1 | 14 | 14 | 15 | 11 | 15 | **69** |
|  | T2 | 15 | 13 | 15 | 11 | 16 | **70** |
|  | T3 | 10 | 11 | 11 | 7 | 8 | **47** |
|  | total | **39** | **38** | **41** | **29** | **39** |  |
| Sofia, NMTI | T1 | 18 | 16 | 17 | 16 | 18 | **85** |
|  | T2 | 19 | 16 | 17 | 17 | 17 | **86** |
|  | T3 | 15 | 15 | 12 | 11 | 14 | **67** |
|  | total | **52** | **47** | **46** | **44** | **49** |  |
| Prague, CU | T1 | 15 | 17 | 18 | 17 | 15 | **82** |
|  | T2 | 17 | 20 | 20 | 18 | 19 | **94** |
|  | T3 | 14 | 8 | 12 | 13 | 11 | **58** |
|  | total | **46** | **45** | **50** | **48** | **45** |  |

Supplementary Table 2. Changes in glycan composition for each center during the observation period. T1 - time point 1, T2 - time point 2, T3 - time point 3, G0 – agalactosylated glycans, G1 – glycans with one galactose, G2 – glycans with two galactoses, S – glycans containing sialic acid, F – fucosylated glycans, B – glycans with bisecting GlcNAc.

| Center | Glycan | T1 - T2 | | T2 - T3 | | |  |
| --- | --- | --- | --- | --- | --- | --- | --- |
|  |  | **Effect** | **Standard Error** | | **Effect** | **Standard Error** | |
| Maastricht | G0 total | -0.09376 | 0.02739 | | 0.00339 | 0.06551 | |
|  | G1 total | -0.08044 | 0.05233 | | 0.15311 | 0.05995 | |
|  | G2 total | -0.01206 | 0.03279 | | 0.10458 | 0.05433 | |
|  | S total | 0.18974 | 0.04667 | | -0.16886 | 0.07197 | |
|  | F total | -0.25468 | 0.04885 | | 0.20851 | 0.05663 | |
|  | B total | -0.01139 | 0.03028 | | 0.04319 | 0.06658 | |
| Copenhagen | G0 total | -0.05246 | 0.02511 | | 0.01887 | 0.03141 | |
|  | G1 total | 0.01383 | 0.04951 | | -0.04827 | 0.05541 | |
|  | G2 total | 0.00420 | 0.02784 | | -0.01528 | 0.03097 | |
|  | S total | 0.09631 | 0.04502 | | 0.00325 | 0.05508 | |
|  | F total | -0.01480 | 0.03986 | | -0.01544 | 0.04156 | |
|  | B total | 0.00278 | 0.02963 | | 0.13487 | 0.03142 | |
| Cambridge | G0 total | -0.05121 | 0.04275 | | 0.05265 | 0.04987 | |
|  | G1 total | 0.00379 | 0.06787 | | -0.00526 | 0.08174 | |
|  | G2 total | -0.01069 | 0.04851 | | 0.05732 | 0.05406 | |
|  | S total | 0.12343 | 0.07236 | | -0.03590 | 0.07540 | |
|  | F total | -0.05964 | 0.05862 | | -0.00970 | 0.07565 | |
|  | B total | 0.06044 | 0.05496 | | 0.02828 | 0.06106 | |
| Heraklion | G0 total | -0.14313 | 0.03520 | | -0.01516 | 0.07860 | |
|  | G1 total | -0.05764 | 0.07309 | | 0.15946 | 0.08265 | |
|  | G2 total | 0.01529 | 0.03147 | | 0.10709 | 0.06283 | |
|  | S total | 0.24505 | 0.06743 | | -0.11952 | 0.09746 | |
|  | F total | -0.10411 | 0.05936 | | -0.05092 | 0.08888 | |
|  | B total | 0.02952 | 0.04522 | | -0.05799 | 0.07736 | |
| Potsdam | G0 total | -0.04097 | 0.02724 | | -0.02016 | 0.03199 | |
|  | G1 total | 0.07039 | 0.07400 | | 0.23101 | 0.07392 | |
|  | G2 total | -0.01183 | 0.03367 | | 0.11144 | 0.03276 | |
|  | S total | 0.02146 | 0.04641 | | -0.18527 | 0.05577 | |
|  | F total | 0.06495 | 0.05190 | | 0.21694 | 0.05631 | |
|  | B total | 0.04063 | 0.04609 | | 0.11232 | 0.04665 | |
| Pamplona | G0 total | -0.05377 | 0.03048 | | 0.00064 | 0.06057 | |
|  | G1 total | 0.01000 | 0.05114 | | -0.07327 | 0.07389 | |
|  | G2 total | 0.00301 | 0.03187 | | 0.03755 | 0.04591 | |
|  | S total | 0.11446 | 0.05186 | | -0.02218 | 0.08869 | |
|  | F total | 0.01736 | 0.04426 | | 0.01752 | 0.09271 | |
|  | B total | -0.00297 | 0.04586 | | 0.11989 | 0.06138 | |
| Sofia | G0 total | -0.04281 | 0.02835 | | 0.05749 | 0.03375 | |
|  | G1 total | 0.12804 | 0.05224 | | -0.01136 | 0.05652 | |
|  | G2 total | 0.01455 | 0.02588 | | -0.01012 | 0.03118 | |
|  | S total | -0.02193 | 0.05109 | | -0.11906 | 0.04753 | |
|  | F total | 0.02272 | 0.03969 | | 0.06495 | 0.05830 | |
|  | B total | -0.02910 | 0.03860 | | 0.03212 | 0.04357 | |
| Prague | G0 total | -0.00642 | 0.04238 | | -0.05344 | 0.03605 | |
|  | G1 total | 0.18081 | 0.04760 | | 0.05079 | 0.05979 | |
|  | G2 total | -0.03777 | 0.04723 | | 0.10287 | 0.03671 | |
|  | S total | -0.06288 | 0.05347 | | 0.00192 | 0.06089 | |
|  | F total | 0.02834 | 0.04512 | | -0.02438 | 0.05051 | |
|  | B total | -0.05559 | 0.03371 | | 0.01318 | 0.05018 | |

Supplementary Table 3. Changes in glycan composition after meta-analysis. G0 – agalactosylated glycans, G1 - glycans with one galactose, G2 – glycans with two galactoses, S – glycans containing sialic acid, F – fucosylated glycans, B – glycans with bisecting GlcNAc.

| Glycan | T1 - T2 | | | | | T2 - T3 | | | | | |
| --- | --- | --- | --- | --- | --- | --- | --- | --- | --- | --- | --- |
|  | **Effect** | **Standard Error** | | **p-value** | **Adjusted**  **p-value** | **Effect** | **Standard Error** | | **p-value** | **Adjusted**  **p-value** | |
| G0 total | -0.0614 | 0.0111 | 3.20E-08 | | 3.84E-07 | 0.0061 | 0.0154 | 0.6932 | | | 0.7981 |
| G1 total | 0.0376 | 0.0312 | 0.2289 | | 0.3434 | 0.0532 | 0.0357 | 0.1361 | | | 0.2333 |
| G2 total | 0.0003 | 0.0116 | 0.9777 | | 0.9777 | 0.0551 | 0.0203 | 0.0066 | | | 0.0199 |
| S total | 0.0842 | 0.0337 | 0.0123 | | 0.0296 | -0.0825 | 0.0277 | 0.0029 | | | 0.0115 |
| F total | -0.0350 | 0.0338 | 0.2997 | | 0.3996 | 0.0564 | 0.0366 | 0.1234 | | | 0.2333 |
| B total | -0.0046 | 0.0134 | 0.7316 | | 0.7981 | 0.0674 | 0.0216 | 0.0018 | | | 0.0108 |


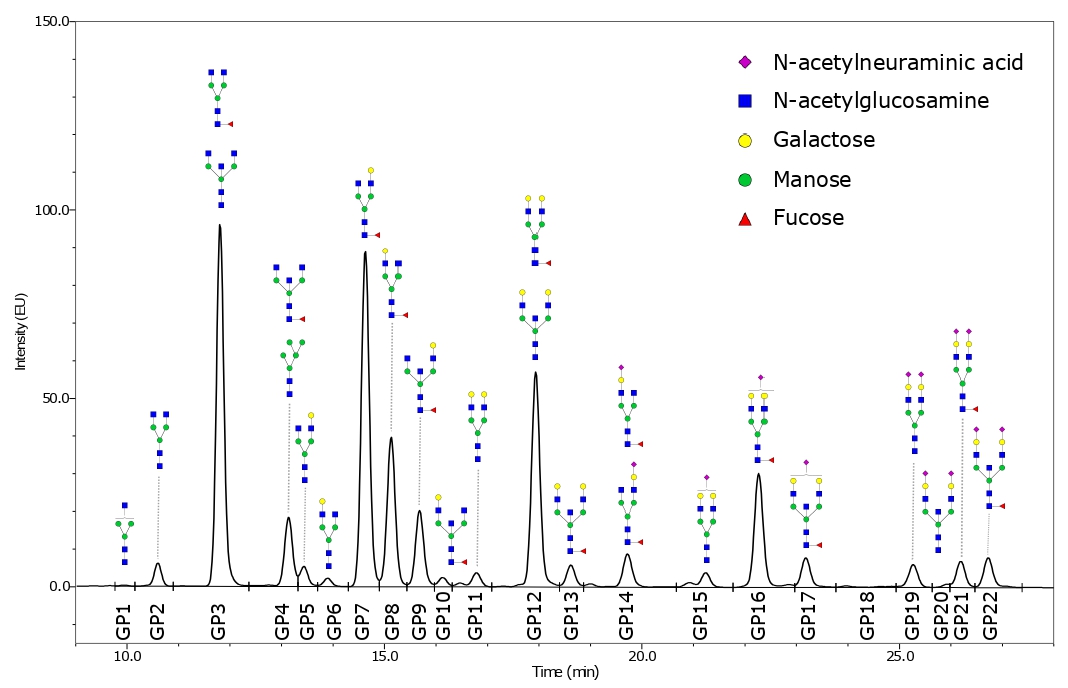


Supplementary Figure 1. Representative HILIC-UPLC-FLR chromatographic profile of the immunoglobulin G RapiFluor-MS labeled N-glycome. Graphic representation of the glycan structures corresponding to each glycan peak (GP). In the case of multiple structures per GP, the upper structure is the major one, and the lower one is minor in abundance. EU = emission units.


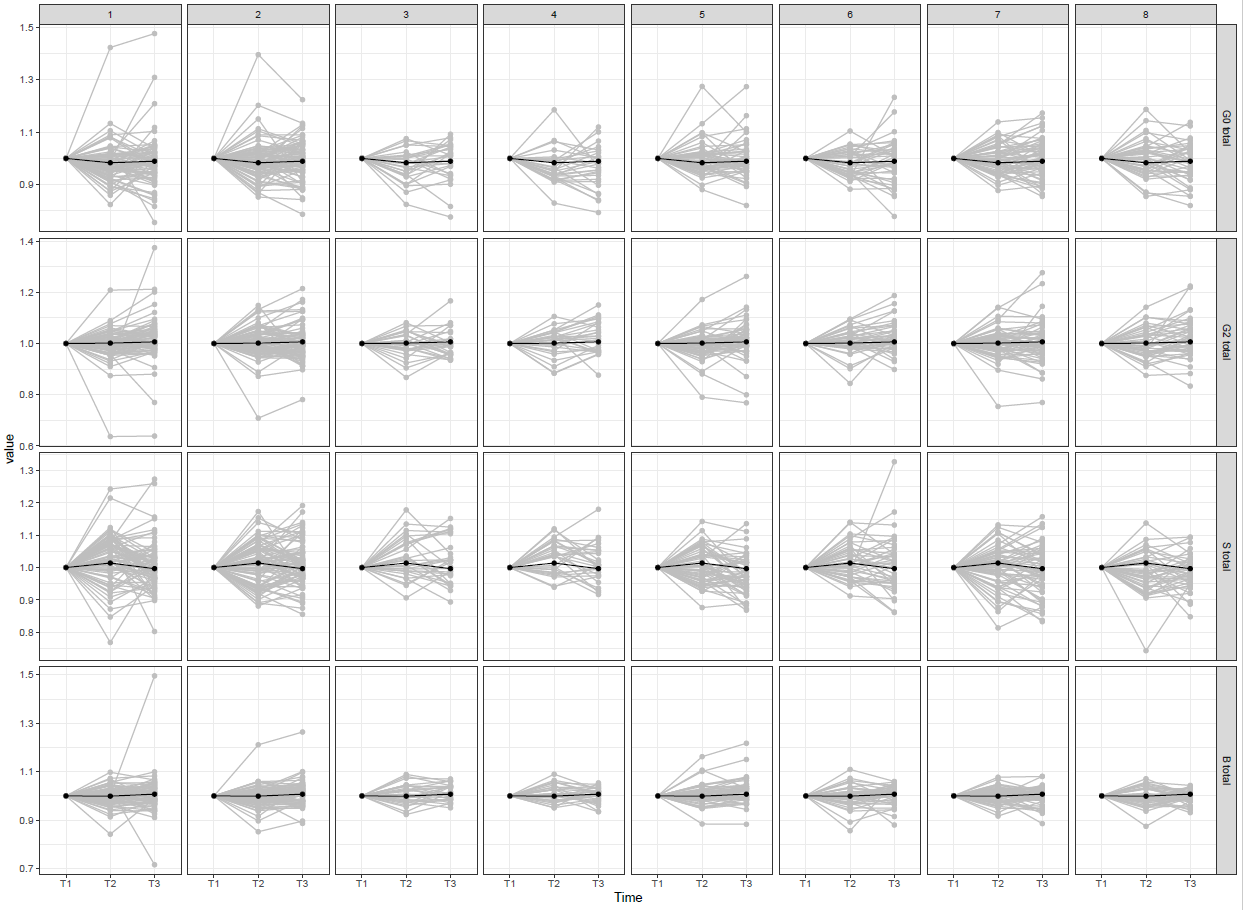


Supplementary Figure 2. IgG glycome composition changes between different centers normalized to the first point. 1- Maastricht, UM, 2 - Copenhagen, RVAU, 3 - Cambridge, HNR, 4 - Heraklion, UoC, 5 - Potsdam, POT, 6 - Pamplona, UNAV, 7 - Sofia, NMTI, 8 - Prague, CU, T1 - time point 1, T2 - time point 2, T3 - time point 3, G0 – agalactosylated glycans, G2 – glycans with two galactoses, S – glycans containing sialic acid, B – glycans with bisecting GlcNAc.


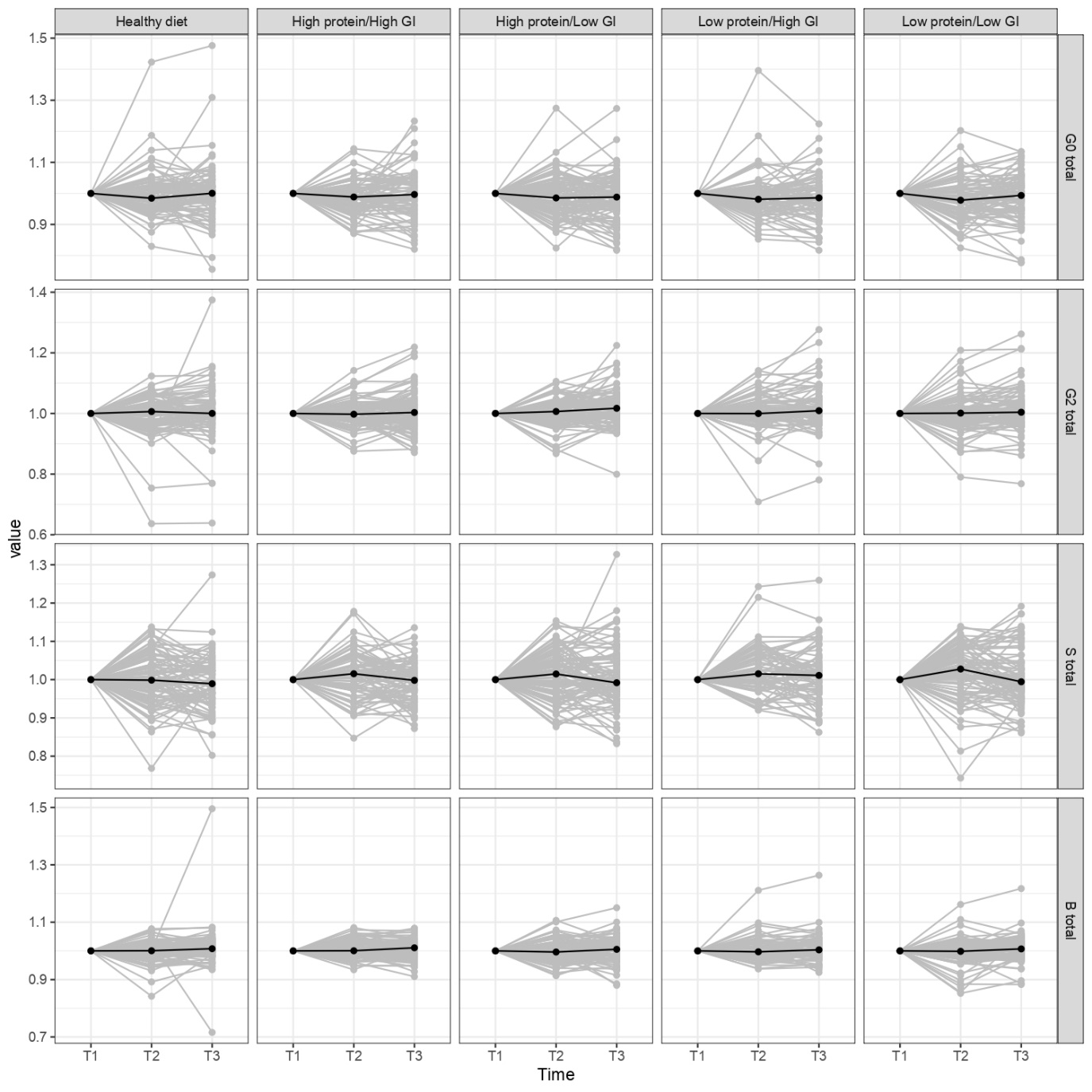


Supplementary Figure 3. IgG glycome composition changes between different diets normalized to the first point. GI – glycaemic index, T1 - time point 1, T2 - time point 2, T3 - time point 3, G0 – agalactosylated glycans, G2 – glycans with two galactoses, S – glycans containing sialic acid, B – glycans with bisecting GlcNAc.
